# The Impact of Thrombophilia on Maternal and Neonatal Outcomes: A Multisystem Analysis of Clinical, Hematological, and Metabolic Parameters

**DOI:** 10.3390/jcm14113665

**Published:** 2025-05-23

**Authors:** Viorela-Romina Murvai, Radu Galiș, Cristina-Maria Macrea, Anca-Florina Tărău-Copos, Marius Daniel Goman, Timea Claudia Ghitea, Anca Huniadi

**Affiliations:** 1 Doctoral School of Biological and Biomedical Sciences, University of Oradea, 1 University Street, 410087 Oradea, Romania; rominna.cuc@gmail.com (V.-R.M.); cristina.ungur10@yahoo.com (C.-M.M.); ancutza_copos@yahoo.com (A.-F.T.-C.); 2 Department of Obstetrics and Gynecology, Emergency County Hospital Bihor, 65 Gheorghe Doja Street, 410169 Oradea, Romania; marius_goman28@yahoo.com; 3 Department of Neonatology, Faculty of Medicine and Pharmacy, University of Oradea, 1 University Street, 410087 Oradea, Romania; raduoradea@yahoo.co.uk; 4 Department of Neonatology, Emergency County Hospital Bihor, 65 Gheorghe Doja Street, 41069 Oradea, Romania; 5 Pharmacy Department, Faculty of Medicine and Pharmacy, University of Oradea, 1 University Street, 410087 Oradea, Romania; 6 Department of Surgical Sciences, Obstetrics and Gynecology, Faculty of Medicine and Pharmacy, University of Oradea, 1 University Street, 410087 Oradea, Romania; ancahuniadi@gmail.com

**Keywords:** thrombophilia, maternal outcomes, neonatal outcomes, proteinuria, coagulation abnormalities, Apgar score

## Abstract

**Background**: Thrombophilia is a prothrombotic disorder that can affect pregnancy outcomes, potentially leading to maternal complications, fetal growth restriction, and adverse perinatal events. However, the precise relationship between thrombophilia and these outcomes remains under investigation, and the impact of hematological, renal, hepatic, and coagulation alterations in thrombophilic pregnancies is not yet fully understood. This study aims to examine the maternal and neonatal consequences of thrombophilia by analyzing key laboratory parameters and perinatal outcomes in affected pregnancies. **Methods**: A retrospective observational study was conducted on 251 pregnant women, divided into thrombophilic (n = 226) and non-thrombophilic (n = 25) groups. Data on maternal demographics, laboratory parameters (hematological, metabolic, renal, hepatic, and coagulation markers), obstetric outcomes, and neonatal characteristics were extracted from medical records. Statistical analysis included *t*-tests, chi-square tests, and Pearson correlation analysis to assess the association between thrombophilia and clinical outcomes. **Results**: Thrombophilic pregnancies were associated with significantly lower fibrinogen levels (*p* = 0.036) and decreased INR (*p* = 0.006), suggesting a hypercoagulable state. Renal function was affected, as evidenced by elevated urea (*p* = 0.012) and creatinine (*p* = 0.009), indicating a predisposition to kidney dysfunction. Neonates from thrombophilic pregnancies exhibited slightly lower Apgar scores at 1 and 5 min, though the differences were not statistically significant (*p* = 0.101, *p* = 0.131). NICU admission rates were comparable between groups (*p* = 0.317), suggesting that thrombophilia may not be a major determinant of neonatal intensive care needs. However, gestational age and birth weight remained the strongest predictors of neonatal vitality (*p* < 0.001), while coagulation abnormalities and renal dysfunction correlated with poorer perinatal outcomes. **Conclusions**: Thrombophilia is associated with altered coagulation profiles, renal dysfunction, and potential risks for maternal complications. While neonatal outcomes were not significantly different, the observed trends suggest the need for enhanced monitoring in thrombophilic pregnancies. Early intervention, thromboprophylaxis, and individualized management strategies may improve maternal and neonatal prognosis. Further research is needed to refine preventive strategies and optimize therapeutic approaches in high-risk pregnancies.

## 1. Introduction

Thrombophilia is a prothrombotic condition that can significantly impact pregnancy outcomes, increasing the risk of maternal complications, placental dysfunction, and adverse neonatal consequences. It is associated with hypercoagulability, which predisposes affected individuals to venous thromboembolism, placental insufficiency, and pregnancy-induced hypertension. Thrombophilic pregnancies have been linked to an increased likelihood of fetal growth restriction, preterm birth, and perinatal morbidity and mortality [1,2]. 

The pathophysiology of thrombophilia in pregnancy is complex and involves vascular endothelial dysfunction, abnormal coagulation activation, and altered placental perfusion, leading to multisystem complications. Laboratory evaluations play a crucial role in diagnosing and managing thrombophilia, helping to assess coagulation status, renal function, and fetal well-being [3,4].

One of the most concerning maternal complications of thrombophilia is coagulation abnormalities, which can result in a hypercoagulable state with an increased risk of thrombosis. This is often reflected in the low International Normalized Ratio (INR), altered activated partial thromboplastin time (APTT), and changes in fibrinogen levels, indicating potential clotting disturbances that may contribute to placental insufficiency and fetal hypoxia [5]. Additionally, elevated serum creatinine levels may indicate impaired renal function, reflecting reduced glomerular filtration due to systemic vasoconstriction and endothelial damage [6].

Renal dysfunction has also been observed in thrombophilic pregnancies, as evidenced by elevated serum urea and creatinine levels, suggesting impaired renal filtration due to microvascular damage and thrombotic events in the renal vasculature [7]. Unlike preeclampsia, proteinuria is not a defining feature of thrombophilia, but vascular injury within the kidney can still contribute to renal dysfunction and pregnancy-related complications [8]. Hepatic involvement in thrombophilia is less well established but may manifest as mild elevations in liver enzymes such as aspartate aminotransferase (AST) and alanine aminotransferase (ALT). These changes may indicate vascular congestion or hepatic microthrombosis, potentially contributing to hepatic dysfunction and increased risk of gestational hypertensive disorders [9].

Inherited maternal thrombophilia has been associated with a wide range of adverse obstetric outcomes, including preeclampsia, placental abruption, and impaired fetal growth secondary to placental dysfunction. In addition to these complications, neonates born to affected mothers may experience increased rates of intrauterine growth restriction (IUGR), preterm birth, and low Apgar scores, largely due to chronic fetal hypoxia. In more severe cases, particularly when neonatal thrombophilia coexists or placental perfusion is markedly compromised, life-threatening thrombotic events such as purpura fulminans, cerebral infarction, or intracranial hemorrhage have also been reported. These adverse outcomes are often associated with poor placental perfusion and thrombotic events in the uteroplacental circulation [10].

Additionally, neonates from thrombophilic pregnancies may require neonatal intensive care unit (NICU) admission due to complications related to low birth weight, prematurity, and respiratory distress [11,12,13].

Neonates born to mothers with thrombophilia are at heightened risk for complications such as respiratory distress syndrome and bronchopulmonary dysplasia. These adverse outcomes are often linked to prematurity and intrauterine growth restriction associated with the maternal condition [14]. A national survey in Romania reported significantly higher NICU admission rates for neonates < 1500 g in level III compared to level II maternity hospitals (66.86 ± 39.14 g vs. 22.87 ± 31.50 g; *p* = 0.002). Additionally, a study from Bihor County found increased stillbirth rates (RR: 1.53, 95% CI, 1.05–2.23), primarily due to intrauterine asphyxia and umbilical cord pathology [15]. These findings emphasize the importance of early intervention and close monitoring of high-risk pregnancies, stressing the need for specialized neonatal care in high-risk pregnancies [16].

This study aims to evaluate the impact of thrombophilia on maternal and neonatal outcomes by analyzing key laboratory markers, coagulation abnormalities, and perinatal complications. Understanding these associations is essential for developing targeted preventive and therapeutic strategies to improve pregnancy outcomes and reduce the risk of maternal and neonatal morbidity.

## 2. Materials and Methods

### 2.1. Study Design and Setting

**Figure 1:**
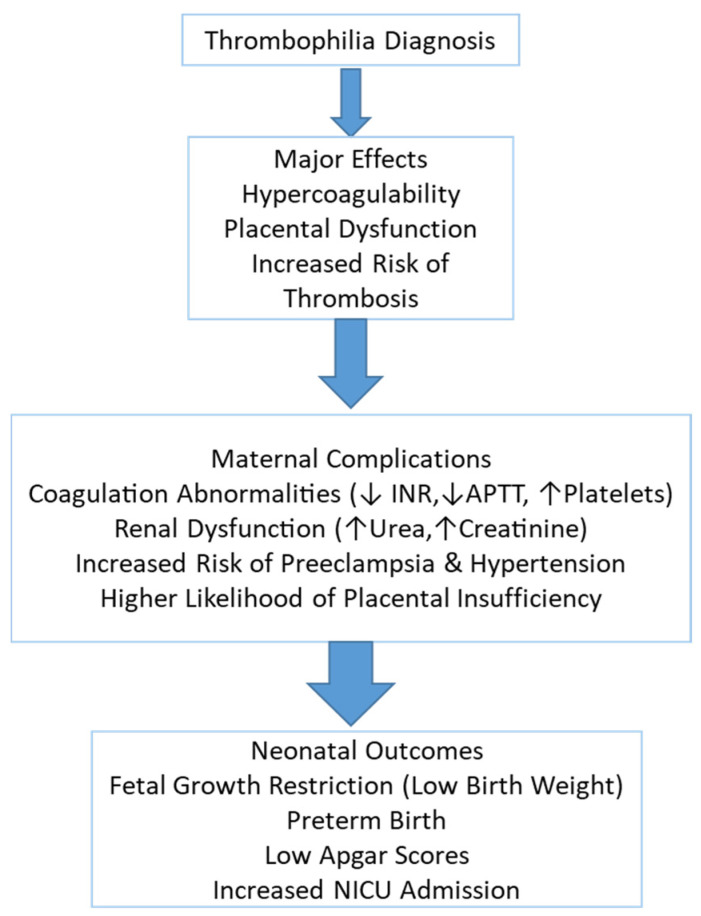
Flowchart.

This study is a retrospective observational analysis conducted on a cohort of pregnant women admitted to Hospital “Spitalul Clinic Județean Bihor” between 1 January 2024 and 31 December 2024. The study aimed to evaluate thrombophilia’s maternal and neonatal impact, analyzing a range of clinical, hematological, metabolic, renal, hepatic, coagulation, and neonatal parameters in preeclamptic and non-preeclamptic pregnancies (Figure 1).

### 2.2. Study Population

This retrospective observational study comprised 251 pregnant women who fulfilled the predefined eligibility criteria. Among them, 226 were identified with inherited thrombophilic mutations, while 25 served as the non-thrombophilic comparison group. The study focused on evaluating pregnancy outcomes in full-term gestations (≥37 weeks), based on the presence or absence of thrombophilia.

1. Thrombophilia Group (Case Group):

The thrombophilia group (n = 226) included patients with the following inherited thrombophilic conditions, diagnosed prior to pregnancy:

Factor V Leiden mutation: 102 cases (45.1%)
Prothrombin G20210A mutation: 54 cases (23.9%)
Protein C deficiency: 29 cases (12.8%)
Protein S deficiency: 21 cases (9.3%)
Antithrombin deficiency: 20 cases (8.8%)

Some patients presented with more than one thrombophilic mutation; however, for classification purposes, each patient was categorized according to their most clinically significant variant. All diagnoses were confirmed through genetic or coagulation assays. Patients who developed preeclampsia during pregnancy were included in this group, provided they had a pre-existing inherited thrombophilia diagnosis.

2. Non-Thrombophilia Group (Control Group):

Pregnant women without a diagnosis of inherited thrombophilia, matched for gestational age and demographic characteristics.

Patients in this group had no history of coagulation disorders or thrombotic events.

Inclusion Criteria

Singleton pregnancies

Gestational age ≥ 37 weeks (full-term pregnancies) at the time of delivery

Confirmed inherited thrombophilia diagnosis prior to pregnancy (for the thrombophilia group).

Availability of complete maternal and neonatal clinical and laboratory data.

Exclusion Criteria

Patients with acquired thrombophilia (e.g., antiphospholipid syndrome)

Chronic hypertension or pre-existing renal/hepatic disease (to distinguish the impact of thrombophilia from pre-existing conditions)

Autoimmune disorders (e.g., systemic lupus erythematosus)

Multiple gestations

Fetal anomalies or congenital malformations

### 2.3. Maternal Demographic and Clinical Data

Age, parity, gestational age at delivery

Presence of metabolic comorbidities (gestational diabetes, gestational hypertension)

Mode of delivery (spontaneous vaginal vs. cesarean section)

Length of hospital stay

### 2.4. Maternal Laboratory Parameters

Hematological markers: Hemoglobin (HGB), hematocrit (HCT), platelet count (PLT), white blood cell count (WBC)

Renal function markers: Urea, creatinine, uric acid, proteinuria

Hepatic function markers: Aspartate aminotransferase (AST), alanine aminotransferase (ALT)

Coagulation parameters: INR, QUICK test, activated partial thromboplastin time (APTT), fibrinogen

Metabolic markers: Fasting blood glucose levels

### 2.5. Neonatal Outcomes

Birth weight

Apgar scores at 1 and 5 min

Neonatal intensive care unit (NICU) admission

### 2.6. Statistical Analysis

Descriptive statistics were used to summarize the data (mean, standard deviation, frequency distributions). Chi-square tests were applied to assess categorical variables. Independent *t*-tests or Mann–Whitney U tests were used to compare continuous variables between groups. Pearson correlation analysis was performed to evaluate relationships between maternal clinical parameters and neonatal outcomes. A *p*-value < 0.05 was considered statistically significant.

### 2.7. Ethical Considerations

The study was conducted in accordance with the Declaration of Helsinki and was approved by the Institutional Ethics Committee of Hospital Approval Number 39007/12 December 2024. Due to the retrospective nature of the study, informed consent was waived, and patient confidentiality was strictly maintained.

## 3. Results

### 3.1. Demographic Description

Table 1 presents statistical data on three variables: Age, Environment, and Gestational Age, based on a total sample size of 251 individuals. Below is an explanation of each variable in paragraph form.

Among the 251 participants, the mean age was 32.3 years (SD = 5.1), ranging from 21 to 46 years, with a distribution close to normal. Urban residents made up 56.2% of the sample, with a fairly balanced urban–rural split. Gestational age averaged 38 weeks (SD = 1.75) but showed a strong left skew, ranging from 28 to 41.5 weeks, indicating a few preterm births in an otherwise full-term population.

The dataset provides insights into the distribution of age, environment, and gestational age in a sample of 251 individuals. The age distribution is fairly normal, with most individuals in their early 30 s. The environment is slightly skewed toward urban living, but the distribution is relatively even. Gestational age shows a strong skew toward higher values, with most pregnancies reaching full term, though some cases involve significantly lower gestational ages.

**Figure 2:**
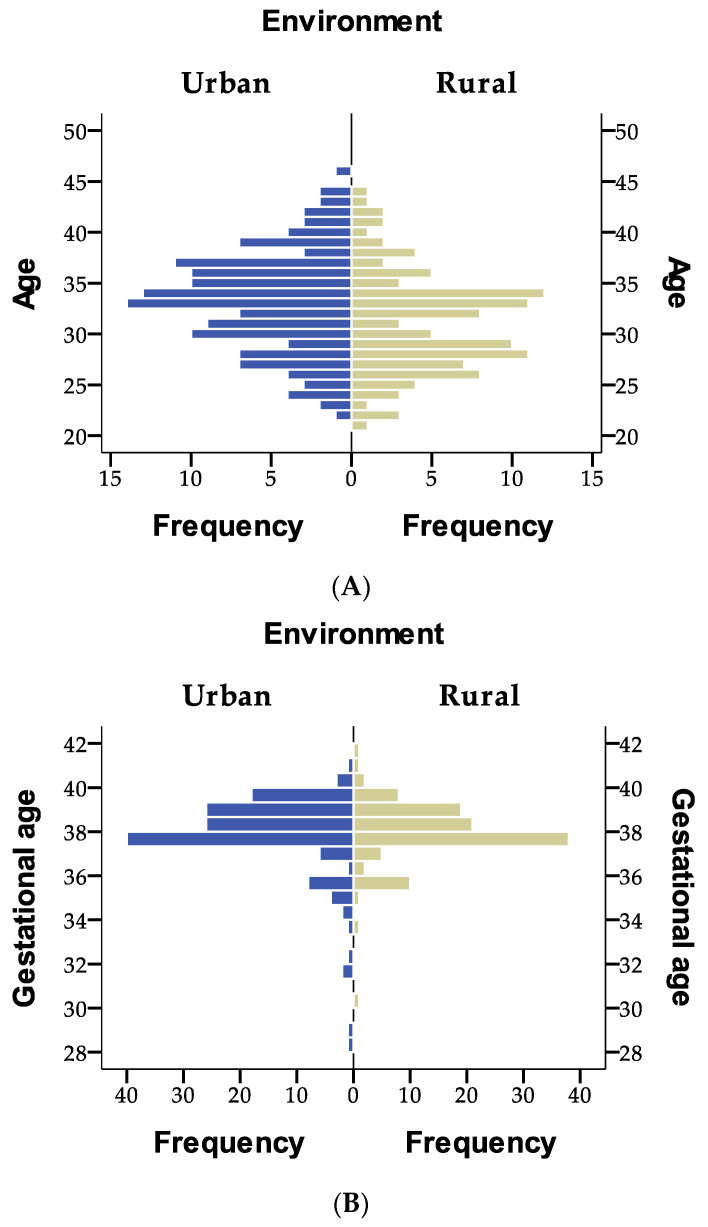
Comparison of age and gestational age distribution by environment (urban vs. rural). (**A**) Age distribution by environment and (**B**) gestational age distribution by environment.

Figure 2A shows the age distribution of individuals based on their environment (urban vs. rural), highlighting a slightly higher concentration of individuals in the urban group around the 30–35 age range. Figure 2B illustrates the distribution of gestational age by environment, with both groups centered around full-term pregnancies (~38–39 weeks) but with some variation, particularly in the rural group.

### 3.2. Impact of Thrombophilia on Maternal and Neonatal Outcomes

Table 2 presents a comparative analysis of key obstetric and neonatal parameters in patients with and without thrombophilia. The statistical evaluation includes mean values, standard deviations, chi-square tests, and their associated asymptotic significance (*p*-values) to assess the relationship between thrombophilia and maternal/neonatal outcomes.

Patients diagnosed with thrombophilia had a similar hospital stay duration, with a mean of 6.08 days (SD = 3.3) compared to 6.26 days (SD = 3.1) in non-thrombophilic patients. The chi-square value of 1.091 and *p*-value of 0.296 indicate that there is no significant association between thrombophilia and prolonged hospitalization in this cohort.

Although thrombophilic patients had a higher number of total pregnancies (gravidity: 2.80 vs. 2.24), they also exhibited a higher number of live births (parity: 2.32 vs. 1.39), resulting in a lower gravidity-to-parity ratio (2.80/2.32 ≈ 1.21) compared to the non-thrombophilic group (2.24/1.39 ≈ 1.61). This suggests that, in our cohort, the relative frequency of pregnancy loss was not higher in the thrombophilic group. This finding may be influenced by the close monitoring and prophylactic anticoagulant therapy administered to thrombophilic patients, potentially contributing to improved pregnancy outcomes despite their elevated baseline risk.

Additionally, neonates from thrombophilic pregnancies had a lower mean birth weight (2760.0 g, SD = 611.4) compared to those from non-thrombophilic pregnancies (3116.7 g, SD = 584.8). The chi-square value of 7.913 and *p*-value of 0.005 indicate that this difference is statistically significant, suggesting a potential association between thrombophilia and fetal growth restriction or placental insufficiency.

These findings indicate that thrombophilia is associated with a history of recurrent pregnancy loss and an increased risk of adverse perinatal outcomes, including reduced neonatal birth weight. While hospitalization duration did not differ significantly between the two groups, the higher gravidity but lower parity in thrombophilic patients highlights the impact of thrombophilia on pregnancy progression and live birth rates. This underscores the need for enhanced surveillance, thromboprophylaxis, and individualized management strategies to improve pregnancy outcomes in thrombophilic women.

The bar chart compares maternal age, gestational age, and neonatal Apgar scores (at 1 and 5 min) between pregnancies with and without thrombophilia. While maternal age is similar between groups, gestational age appears slightly lower in thrombophilic pregnancies, indicating a potential higher risk of preterm birth. Additionally, Apgar scores at 1 and 5 min are lower in the thrombophilia group, suggesting compromised neonatal adaptation and an increased likelihood of perinatal complications in these pregnancies.

**Figure 3:**
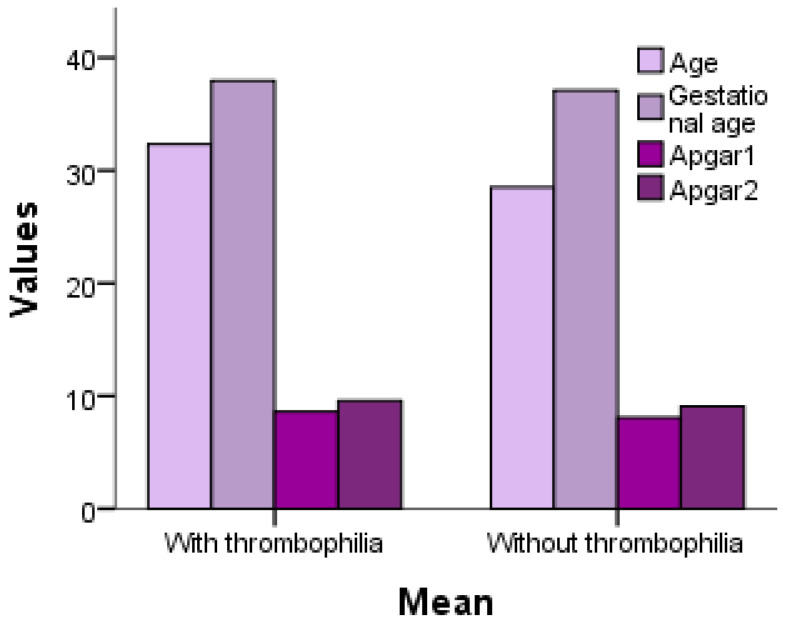
Comparison of maternal age, gestational age, and neonatal Apgar scores in thrombophilic and non-thrombophilic pregnancies.

Thecomparison of maternal age, gestational age, and neonatal Apgar scores in thrombophilic and non-thrombophilic pregnancies is presented in Figure 3.

### 3.3. Maternal and Neonatal Characteristics in Thrombophilic and Non-Thrombophilic Pregnancies

Among patients with thrombophilia (n = 226), 96.0% (n = 217) did not have gestational diabetes, while 4.0% (n = 9) were diagnosed with it. In contrast, none of the non-thrombophilic patients (n = 25) were affected by gestational diabetes.

Among patients with thrombophilia, 87.7% (n = 242) did not have gestational diabetes, while 3.3% (n = 9) were diagnosed with it. In contrast, among patients without thrombophilia, all 25 individuals (100%) had no diagnosis of gestational diabetes. This reflects the known metabolic risks associated with thrombophilia and suggests a distinct profile of glucose regulation in these patients. The chi-square test value of 176.3 and a *p*-value of *p* < 0.001 indicate a highly significant association between thrombophilia and gestational diabetes, suggesting that patients with thrombophilia exhibit a distinct metabolic profile with an increased risk of glucose regulation abnormalities.

In the thrombophilic group (n = 226), 81.9% (n = 185) did not have gestational hypertension, while 11.1% (n = 25) were diagnosed with it. In the non-thrombophilic group (n = 25), 100% (n = 25) did not have gestational hypertension. The chi-square analysis reveals two statistically significant associations: a strong overall relationship (χ^2^ = 163.2, *p* < 0.001) and an additional significant difference for hypertensive patients (χ^2^ = 22.15, *p* < 0.001). These findings confirm that thrombophilia is significantly associated with gestational hypertension, reinforcing its role as a prothrombotic and vascular risk factor during pregnancy.

Among non-thrombophilic patients, 76.4% (n = 211) delivered via cesarean section, while 14.5% (n = 40) had a spontaneous vaginal birth. In contrast, in the thrombophilic group, 6.2% (n = 17) had a cesarean section, while 2.9% (n = 8) had a vaginal delivery. The chi-square test values (χ^2^ = 165.1 for cesarean section and χ^2^ = 21.33 for spontaneous birth, both *p* < 0.001) indicate a highly significant relationship between thrombophilia and mode of delivery. These results suggest that thrombophilia is strongly associated with an increased likelihood of cesarean delivery, possibly due to higher obstetric risks, including placental dysfunction and fetal distress.

In non-thrombophilic pregnancies, 40.9% (n = 113) of newborns were male, while 50.0% (n = 138) were female. In the thrombophilia group, 4.3% (n = 12) of newborns were male, and 4.7% (n = 13) were female. The chi-square tests indicate strong associations between neonatal sex and thrombophilia (χ^2^ = 81.61, *p* < 0.001 for males; χ^2^ = 103.5, *p* < 0.001 for females), suggesting potential differences in fetal sex distribution among thrombophilic pregnancies. However, the biological mechanisms underlying this association remain unclear and warrant further investigation.

This analysis demonstrates that thrombophilia is significantly associated with gestational diabetes, gestational hypertension, cesarean delivery, and neonatal sex distribution. The data suggest that thrombophilia contributes to an increased risk of hypertensive disorders and a higher probability of requiring cesarean section, likely due to maternal and fetal complications (Table 3). Additionally, the observed differences in fetal sex distribution among thrombophilic pregnancies may reflect an underlying trend, although further research is needed to determine its clinical significance.

### 3.4. Laboratory Abnormalities and Systemic Impact of Preeclampsia 

This table presents a comparative analysis of hematological, renal, hepatic, and coagulation parameters in patients with and without thrombophilia. The analysis includes mean values, standard deviations, chi-square tests, and *p*-values, providing insight into the impact of thrombophilia on various laboratory markers.

#### 3.4.1. Hematological Parameters

White Blood Cell Count (WBC): The mean WBC count was slightly lower in thrombophilic patients (11.70 × 10^9^/L, SD = 3.1) compared to non-thrombophilic patients (11.83 × 10^9^/L, SD = 2.8). However, this difference was not statistically significant (χ^2^ = 0.273, *p* = 0.601), suggesting that leukocyte levels are not significantly affected by thrombophilia.

Hemoglobin (HGB): The mean hemoglobin level was 11.42 g/dL (SD = 1.3) in thrombophilic patients, significantly lower than in non-thrombophilic patients (14.67 g/dL, SD = 29.1). The statistical significance (χ^2^ = 8.233, *p* = 0.004) indicates that thrombophilia is associated with lower hemoglobin levels, potentially reflecting a higher risk of anemia in affected pregnancies.

Hematocrit (HCT): Similarly, hematocrit levels were lower in thrombophilic pregnancies (34.80%, SD = 3.9) compared to non-thrombophilic pregnancies (35.95%, SD = 3.5), but this difference was not statistically significant (χ^2^ = 1.506, *p* = 0.220).

Platelet Count (PLT): Platelet counts were higher in thrombophilic patients (253.0 × 10^9^/L, SD = 89.8) compared to non-thrombophilic patients (233.7 × 10^9^/L, SD = 62.8). However, this difference did not reach statistical significance (χ^2^ = 1.110, *p* = 0.292), suggesting that thrombophilia does not significantly impact platelet levels in this cohort.

#### 3.4.2. Renal Function Markers

Blood Urea (Urea): Thrombophilic patients had significantly higher urea levels (18.44 mg/dL, SD = 0) compared to non-thrombophilic patients (16.67 mg/dL, SD = 4.7), with a statistically significant difference (χ^2^ = 6.323, *p* = 0.012). This suggests impaired renal function, possibly due to vascular alterations associated with thrombophilia.

Serum Creatinine: Creatinine levels were markedly elevated in thrombophilic patients (3.07 mg/dL, SD = 12.4) compared to non-thrombophilic patients (0.62 mg/dL, SD = 0.1), with a statistically significant difference (χ^2^ = 6.874, *p* = 0.009). This suggests potential kidney dysfunction or altered renal clearance mechanisms in thrombophilic pregnancies.

Uric Acid: Thrombophilic patients exhibited lower mean serum uric acid levels (4.01 mg/dL, SD = 0.7) compared to non-thrombophilic patients (4.40 mg/dL, SD = 1.2). However, this difference was not statistically significant (χ^2^ = 2.452, *p* = 0.117), indicating no strong association between thrombophilia and uric acid levels in this cohort.

#### 3.4.3. Liver Enzymes

Aspartate Aminotransferase (AST): AST levels were slightly higher in thrombophilic patients (24.20 U/L, SD = 28.3) compared to non-thrombophilic patients (22.60 U/L, SD = 36.6), but this difference was not statistically significant (χ^2^ = 1.312, *p* = 0.252), suggesting no clear association between thrombophilia and hepatic dysfunction.

Alanine Aminotransferase (ALT): ALT levels were lower in thrombophilic patients (19.16 U/L, SD = 18.0) compared to non-thrombophilic patients (21.89 U/L, SD = 45.2), with a non-significant difference (χ^2^ = 0.031, *p* = 0.861). This further supports the lack of a strong relationship between thrombophilia and liver dysfunction in this study.

#### 3.4.4. Coagulation Parameters

Fibrinogen: Although fibrinogen levels were lower in thrombophilic patients (479.9 mg/dL, SD = 87.3) compared to non-thrombophilic patients (528.5 mg/dL, SD = 118.7), this difference was statistically significant (χ^2^ = 4.413, *p* = 0.036). This suggests a potential impact of thrombophilia on coagulation balance, which may contribute to thrombotic risks.

International Normalized Ratio (INR): INR levels were significantly lower in thrombophilic patients (0.94, SD = 0.7) compared to non-thrombophilic patients (0.98, SD = 0.1), with a *p*-value of 0.006 (χ^2^ = 7.604). This finding indicates a hypercoagulable state in thrombophilic pregnancies, increasing the risk of thrombotic complications.

Prothrombin Time (QUICK test): The QUICK test was slightly lower in thrombophilic patients (10.6 s, SD = 0.6) compared to non-thrombophilic patients (10.7 s, SD = 1.4), but this difference was not statistically significant (χ^2^ = 0.031, *p* = 0.860).

Activated Partial Thromboplastin Time (APTT): APTT values were lower in thrombophilic patients (25.6 s, SD = 1.5) compared to non-thrombophilic patients (26.7 s, SD = 2.8), with a statistically significant difference (χ^2^ = 5.663, *p* = 0.017). This further supports a prothrombotic tendency in thrombophilic pregnancies.

The hematological, renal, hepatic, and coagulation alterations in thrombophilic and non-thrombophilic pregnancies are presented in Table 4.

### 3.5. Multisystemic Complications and Therapeutic Approaches in Thrombophilic Pregnancies

This table presents a comparative analysis of key urinary, hematological, infectious, and therapeutic parameters in patients with and without thrombophilia. The statistical evaluation includes mean values, standard deviations, absolute counts, percentage distributions, chi-square test values, and *p*-values, providing insights into the associations between thrombophilia and these clinical factors.

#### 3.5.1. Proteinuria

Proteinuria, a marker of renal involvement, was lower in thrombophilic patients, with a mean value of 9.16 mg/dL (SD = 16.05) compared to 18.48 mg/dL (SD = 37.82) in non-thrombophilic patients. The chi-square value of 12.574 and *p*-value of 0.000 indicate a highly significant difference, suggesting that thrombophilia is not associated with severe proteinuria, unlike other pregnancy-related disorders such as preeclampsia.

#### 3.5.2. Urinary Tract Infections (UTIs)

Among non-thrombophilic patients, 89.1% (n = 246) did not have a urinary tract infection (UTI), while 1.8% (n = 5) were affected.

In thrombophilic patients, 9.1% (n = 25) did not have a UTI, and 0% had an infection.

The statistical analysis (χ^2^ = 180.180, *p* = 0.001) indicates a significant difference, but the absence of UTIs in thrombophilic patients may reflect differences in detection rates, sample size limitations, or the impact of thrombophilia-related vascular changes on infection susceptibility.

#### 3.5.3. Blood Transfusion

Blood transfusions were more frequent in non-thrombophilic patients (1.4%, n = 4) compared to 0% in thrombophilic patients, with a significant association (χ^2^ = 181.180, *p* = 0.001). While unexpected, this finding may indicate that non-thrombophilic patients required transfusions for other obstetric complications, whereas thrombophilic patients may have been under closer monitoring with proactive anticoagulant therapy to prevent severe hemorrhagic events.

#### 3.5.4. Complications

Among thrombophilic patients, 86.6% (n = 239) had no complications, while 4.3% (n = 12) experienced complications.

In non-thrombophilic patients, 8.7% (n = 24) had no complications, while 0.4% (n = 1) experienced complications.

The chi-square test values (χ^2^ = 175.7, *p* = 0.000 for overall complications and χ^2^ = 9.308, *p* = 0.002 for specific complications) indicate a statistically significant association between thrombophilia and overall pregnancy complications. However, the distribution within the thrombophilic group does not strongly differentiate from non-thrombophilic patients, suggesting that complication rates may depend on additional risk factors rather than thrombophilia alone.

#### 3.5.5. Vaginal Infections

Vaginal infections were significantly associated with thrombophilia (χ^2^ = 73.081, *p* = 0.001).

*Candida* sp. was the most common infection in non-thrombophilic patients (17.4%, n = 48) but was significantly lower in thrombophilic patients (0.4%, n = 1, χ^2^ = 45.082, *p* = 0.001).

*E. coli* infections were observed in 7.6% (n = 21) of non-thrombophilic cases, while only 0.7% (n = 2) of thrombophilic cases were affected (χ^2^ = 15.696, *p* = 0.001).

*Streptococcus* sp. infections were present in 1.8% (n = 5) of non-thrombophilic patients, while no cases were reported in thrombophilic patients.

*Klebsiella* sp. infections occurred in 2.2% (n = 6) of non-thrombophilic cases and were absent in thrombophilic patients.

Other infections were more frequent in non-thrombophilic patients (6.9%, n = 19) than in thrombophilic patients (0%, n = 0).

Multiple infections were observed more often in non-thrombophilic patients (9.4%, n = 26) compared to thrombophilic cases (0%, n = 0).

The associations between thrombophilia, renal dysfunction, infections, comorbidities, and treatment strategies is presented in Table 5.

### 3.6. Impact of Thrombophilia on Neonatal Health and Immediate Postnatal Adaptation

Table 6 presents a comparative analysis of neonatal outcomes in thrombophilic and non-thrombophilic pregnancies, focusing on Apgar scores at 1 and 5 min and the need for neonatal intensive care unit (NICU) admission. The analysis includes mean values, standard deviations, absolute counts, chi-square test values, and *p*-values to assess the impact of thrombophilia on neonatal health.

#### 3.6.1. Apgar Score at 1 Minute (Apgar 1)

Newborns from non-thrombophilic pregnancies had a mean Apgar score of 8.6 (SD = 1.4), while those from thrombophilic pregnancies had a slightly lower mean score of 8.1 (SD = 2.1).

The chi-square test (χ^2^ = 2.686, *p* = 0.101) indicates that this difference is not statistically significant, suggesting that thrombophilia does not have a major impact on the newborn’s immediate postnatal condition in this sample. However, the slightly lower Apgar scores in thrombophilic pregnancies may indicate a trend toward perinatal distress, possibly due to placental insufficiency or fetal compromise.

#### 3.6.2. Apgar Score at 5 Minutes (Apgar 2)

By 5 min, the Apgar score remained lower in thrombophilic newborns (9.1, SD = 2.1) compared to non-thrombophilic newborns (9.6, SD = 1.5).

The chi-square test (χ^2^ = 2.280, *p* = 0.131) shows that the difference is not statistically significant, although the trend toward lower neonatal vitality in thrombophilic pregnancies persists.

While newborns from thrombophilic pregnancies tend to recover over time, the slight reduction in Apgar scores may reflect increased susceptibility to perinatal complications, delayed transition to extrauterine life, or mild neonatal hypoxia.

#### 3.6.3. Neonatal Intensive Care Unit (NICU) Admission

Most newborns (89.5%, n = 247) from non-thrombophilic pregnancies did not require NICU admission, compared to 7.6% (n = 21) of newborns from thrombophilic pregnancies.

NICU admission was required in 1.4% (n = 4) of both thrombophilic and non-thrombophilic neonates.

The chi-square test (χ^2^ = 1.000, *p* = 0.317) indicates that this difference is not statistically significant, suggesting that thrombophilia does not have a strong independent effect on NICU admission rates in this dataset. However, the higher proportion of thrombophilic newborns in the NICU group may indicate that some cases required additional medical support, even if the association was not statistically confirmed.

**Figure 4:**
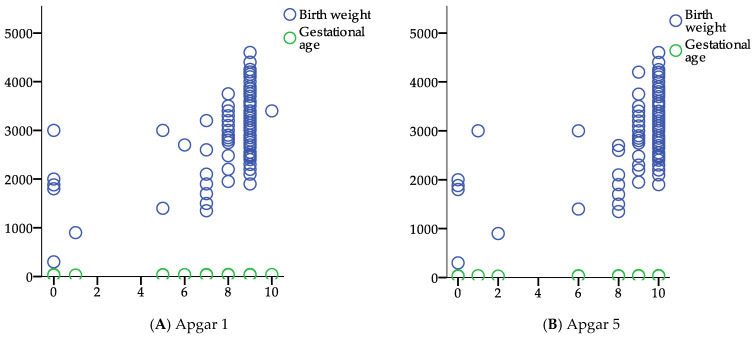
Scatter plot of Apgar scores (1 and 5 min) in relation to birth weight and gestational age.

The scatter plot figure illustrates the relationships between Apgar scores (at 1 and 5 min) and various maternal and neonatal parameters, including birth weight, INR, proteinuria, and gestational age (Figure 4). Birth weight (blue circles) shows a positive trend with higher Apgar scores, indicating better neonatal outcomes in infants with higher birth weights. INR, proteinuria, and gestational age appear more evenly distributed, suggesting weaker correlations with Apgar scores. These findings emphasize that lower Apgar scores are associated with lower birth weights, which are more common in thrombophilic pregnancies.

### 3.7. Maternal Factors Influencing Neonatal Vitality and Postnatal Adaptation

Table 7 presents the Pearson correlation coefficients (r) and their associated *p*-values (*p*), analyzing the relationship between various maternal and neonatal parameters with Apgar scores at 1 min (Apgar 1) and 5 min (Apgar 5). The correlation values indicate the strength and direction of association, with positive values suggesting a direct correlation (higher values of the independent variable are associated with higher Apgar scores), while negative values suggest an inverse correlation (higher values of the independent variable are associated with lower Apgar scores).

#### 3.7.1. Positive Correlations with Apgar Scores

Gestational age showed a strong positive correlation with both Apgar 1 (r = 0.568, *p* = 0.000) and Apgar 2 (r = 0.561, *p* = 0.000). This indicates that higher gestational age is associated with better neonatal condition at birth, reinforcing the importance of fetal maturity in perinatal outcomes.

Birth weight also had a moderate positive correlation with both Apgar 1 (r = 0.500, *p* = 0.000) and Apgar 2 (r = 0.496, *p* = 0.000). This suggests that higher birth weight is associated with better neonatal adaptation, which aligns with known risks of low birth weight, such as respiratory distress and metabolic instability in neonates.

#### 3.7.2. Negative Correlations with Apgar Scores

The International Normalized Ratio (INR) showed a weak but significant negative correlation with both Apgar 1 (r = −0.239, *p* = 0.000) and Apgar 2 (r = −0.214, *p* = 0.001). This suggests that higher INR values (indicating prolonged clotting time) are associated with lower Apgar scores, possibly due to coagulation abnormalities affecting placental function or fetal well-being.

The prothrombin time (QUICK test) also had a weak negative correlation with Apgar 1 (r = −0.217, *p* = 0.001) and Apgar 2 (r = −0.195, *p* = 0.002), further suggesting a potential impact of coagulation status on neonatal outcomes.

The activated partial thromboplastin time (APTT) had a slightly stronger negative correlation with Apgar 1 (r = −0.283, *p* = 0.000) and Apgar 2 (r = −0.262, *p* = 0.000), indicating that prolonged clotting time is associated with poorer neonatal condition, possibly due to vascular complications affecting fetal oxygenation.

Proteinuria, a hallmark of preeclampsia, was negatively correlated with both Apgar 1 (r = −0.261, *p* = 0.000) and Apgar 2 (r = −0.245, *p* = 0.000). This supports the association between maternal renal dysfunction, fetal distress, and adverse neonatal outcomes.

Glucose levels had a weak negative correlation with Apgar 2 (r = −0.125, *p* = 0.048), indicating a borderline significant relationship between maternal glucose levels and neonatal adaptation. Although the association is weak, maternal glucose imbalances may contribute to neonatal metabolic instability.

This analysis highlights that gestational age and birth weight are the strongest positive predictors of better neonatal outcomes, while coagulation abnormalities (INR, QUICK, APTT), proteinuria, and glucose levels have a negative impact on neonatal Apgar scores. These findings reinforce the importance of optimal maternal health, proper coagulation function, and reduced proteinuria in improving perinatal outcomes.

## 4. Discussion

Thrombophilia is a prothrombotic disorder that can significantly impact maternal health, placental function, and neonatal outcomes [17]. Our study analyzed various clinical parameters to assess these effects, and our findings align with the existing literature, reinforcing the complex nature of thrombophilia in pregnancy.

Our study identified coagulation abnormalities, including altered INR and APTT values, in thrombophilic patients. These findings are consistent with previous research demonstrating a hypercoagulable state in thrombophilic pregnancies, which increases the risk of thromboembolic events, placental insufficiency, and gestational hypertensive disorders [18]. The significantly lower INR values observed in thrombophilic patients (*p* = 0.006) suggest an increased risk of clot formation, which may contribute to vascular complications and placental dysfunction. Despite this, no cases of clinically manifest thrombotic complications, such as deep vein thrombosis or pulmonary embolism, were recorded in our cohort. This finding may be attributed to the implementation of prophylactic anticoagulation with low molecular weight heparin (enoxaparin), in line with current clinical guidelines, and to rigorous antenatal monitoring. These preventive measures likely contributed to the absence of overt thrombotic events, highlighting the importance of tailored surveillance and management in pregnancies affected by inherited thrombophilia.

Our findings indicate higher serum urea and creatinine levels in thrombophilic patients compared to non-thrombophilic controls (*p* = 0.012, *p* = 0.009, respectively), suggesting impaired renal function. Although proteinuria was not as pronounced as in preeclamptic pregnancies, vascular alterations and microthrombotic events in renal circulation may contribute to reduced glomerular filtration and kidney dysfunction in thrombophilic women [19].

An isolated elevation of serum creatinine during pregnancy, in the absence of proteinuria, does not necessarily indicate renal dysfunction. Given the physiological increase in glomerular filtration rate during gestation, even slight elevations may appear abnormal. Such variations can also result from transient factors like dehydration or laboratory variability. In our cohort, the lack of proteinuria and other clinical signs of renal impairment suggests that the elevated creatinine values were not indicative of true nephropathy.

These results are consistent with studies suggesting that thrombophilic disorders can affect renal perfusion, leading to increased renal vascular resistance and potential kidney injury [20]. 

Our study found no significant differences in AST and ALT levels between thrombophilic and non-thrombophilic pregnancies, suggesting minimal hepatic involvement. However, previous research has suggested that hepatic microvascular thrombosis and vascular congestion in thrombophilic pregnancies may contribute to mild liver dysfunction [21]. 

Contrary to expectations based on the pathophysiology of thrombophilia, our data showed a lower pregnancy-to-live birth ratio in the thrombophilic group. This could reflect the benefits of early diagnosis and individualized management strategies, such as thromboprophylaxis with enoxaparin or aspirin, which may help mitigate the risk of implantation failure or early pregnancy loss. These findings suggest that proactive management may enhance reproductive outcomes in thrombophilic patients, a hypothesis that warrants further prospective investigation.

Although HELLP syndrome (Hemolysis, Elevated Liver Enzymes, Low Platelets) is more commonly associated with preeclampsia, its occurrence in thrombophilic pregnancies cannot be ruled out, especially in cases of severe coagulopathy or endothelial dysfunction [22,23]. 

Our study found that thrombophilia was associated with lower birth weights and a slight reduction in gestational age, though the differences were not statistically significant. These findings align with research showing that placental thrombosis and impaired vascular remodeling in thrombophilic pregnancies can lead to fetal growth restriction [24,25].

While the differences in Apgar scores at 1 and 5 min between thrombophilic and non-thrombophilic newborns were not statistically significant, the trend toward lower scores in thrombophilic pregnancies suggests an increased risk of perinatal distress [26].

Several maternal factors in thrombophilic pregnancies have been associated with lower Apgar scores and adverse neonatal outcomes:

Placental Insufficiency: Inadequate placental perfusion due to microvascular thrombi can lead to chronic fetal hypoxia, increasing the risk of low Apgar scores and neonatal morbidity [27,28]. Hypercoagulability and Fetal Growth Restriction: The prothrombotic state in thrombophilia can impair nutrient and oxygen exchange, contributing to intrauterine growth restriction (IUGR) and neonatal complications [29,30].

Increased Risk of Preterm Birth: Previous studies have linked thrombophilic pregnancies with a higher likelihood of preterm delivery, which can further affect neonatal Apgar scores and the need for NICU admission [31,32].

Our study found no significant difference in NICU admissions between thrombophilic and non-thrombophilic newborns (*p* = 0.317). However, previous research has indicated that thrombophilia is associated with a higher risk of neonatal complications, particularly in cases of severe placental insufficiency or maternal coagulopathy [33,34].

However, no thrombotic events such as purpura fulminans, cerebral infarction, vitreous hemorrhage, or cerebral hemorrhage were observed among the neonates in our cohort. This may be attributed to the absence of severe maternal thrombotic manifestations, the effective antenatal management of thrombophilic pregnancies—including prophylactic anticoagulation with low molecular weight heparin—and the lack of additional neonatal risk factors that could predispose to thrombotic complications. The indications for NICU admission were primarily related to non-thrombotic causes, such as prematurity, neonatal pneumonia, or impaired postnatal adaptation.

While our study did not identify a strong link, continued neonatal surveillance in thrombophilic pregnancies remains crucial, as even mild clotting disturbances can compromise placental function and fetal well-being [4,35].

Our findings reinforce the importance of individualized management strategies in thrombophilic pregnancies:

Coagulation Monitoring: Regular assessment of INR, APTT, and fibrinogen levels is essential for preventing maternal thromboembolic events and placental dysfunction [36].

Renal Function Evaluation: Given the increased urea and creatinine levels observed in thrombophilic pregnancies, renal function monitoring should be integrated into routine care.

Fetal Growth Surveillance: Due to the association between thrombophilia, placental insufficiency, and fetal growth restriction, serial ultrasound and Doppler flow studies should be considered to optimize neonatal outcomes.

Thromboprophylaxis and Antiplatelet Therapy: The use of low-molecular-weight heparin (LMWH) and aspirin prophylaxis may play a crucial role in reducing thrombotic complications and improving placental perfusion in high-risk pregnancies [37,38,39].

### 4.1. Limitation

This study has several limitations that should be acknowledged.

Retrospective Study Design: As a retrospective observational study, the reliance on previously recorded data may introduce bias due to incomplete or missing information. Additionally, the study design limits the ability to establish causal relationships between thrombophilia and pregnancy outcomes.

Single-Center Data: The study was conducted in a single healthcare institution, which may limit the generalizability of findings to diverse populations with different genetic and environmental risk factors.

Sample Size: The number of thrombophilic cases was relatively small, which may have reduced the statistical power for detecting subtle but clinically significant associations.

Lack of Long-Term Follow-Up: This study focused on immediate neonatal outcomes (Apgar scores, NICU admissions, and birth weight) without longitudinal follow-up to assess long-term neurodevelopmental or cardiovascular consequences in children born to thrombophilic mothers.

Unmeasured Confounding Factors: Certain maternal factors, such as nutritional status, socioeconomic background, and genetic predisposition, were not accounted for, potentially influencing study findings.

### 4.2. Future Research Directions

To overcome these limitations, future studies should do the following:

Conduct prospective multicenter studies with larger sample sizes to enhance statistical power and generalizability.

Investigate the long-term effects of thrombophilia on both maternal and neonatal health, including neurodevelopmental outcomes and cardiovascular risks in children.

Assess the effectiveness of targeted anticoagulant and antiplatelet therapies, such as LMWH and aspirin, in improving maternal vascular health and fetal outcomes.

## 5. Conclusions

This study highlights the multifactorial impact of thrombophilia on maternal and neonatal health, demonstrating significant associations between hematological, renal, hepatic, coagulation, and infectious parameters with adverse pregnancy outcomes. The findings reinforce the systemic nature of thrombophilia and its potential to contribute to maternal complications and neonatal morbidity.

Thrombophilic pregnancies were associated with prolonged hospitalization and an increased need for medical interventions, reflecting the higher burden of maternal morbidity. Renal dysfunction, evidenced by elevated urea and creatinine levels, suggests impaired kidney function, which may be linked to vascular abnormalities associated with thrombophilia. While hepatic enzyme levels (AST, ALT) did not show a significant increase, coagulation abnormalities (INR, APTT, fibrinogen) suggested an underlying hypercoagulable state, which may increase the risk of thrombotic events during pregnancy and postpartum.

Thrombophilia was significantly associated with lower birth weight, potentially indicating placental insufficiency as a major contributing factor to fetal growth restriction. While Apgar scores at 1 and 5 min were lower in thrombophilic pregnancies, the difference was not statistically significant, suggesting that although thrombophilia may contribute to perinatal distress, the overall neonatal impact remains moderate in this cohort. NICU admission rates were comparable between thrombophilic and non-thrombophilic pregnancies, implying that other maternal risk factors may play a more substantial role in determining neonatal morbidity.

These findings emphasize the importance of early identification and close monitoring of thrombophilic pregnancies, particularly in women with coagulation disorders, vascular complications, or previous pregnancy-related thrombotic events. Preventive strategies, such as thromboprophylaxis (e.g., enoxaparin) and aspirin prophylaxis, could play a key role in reducing maternal and neonatal complications. Optimized antenatal surveillance, including Doppler ultrasound to assess placental function and serial fetal growth monitoring, may help mitigate fetal growth restriction and perinatal distress.

### Future Directions

Further research should focus on long-term follow-up of both mothers and neonates to assess the lasting consequences of thrombophilia on pregnancy outcomes and child development. Additionally, studies evaluating the efficacy of tailored anticoagulant and antiplatelet therapies in thrombophilic pregnancies could provide more targeted management strategies. Expanding the study to include a larger and more diverse population would enhance the understanding of risk factors, genetic predispositions, and potential therapeutic interventions to optimize pregnancy outcomes in women with thrombophilia.

## Figures and Tables

**Table 1:** Descriptive statistics of maternal age, environment, and gestational age in the study population.

Parameters		Age	Environment		Gestational Age
N	Valid	251			
	Missing	0	0		0
Mean (N)		32.3	Urban	Rural	37.9
			141	110	
SD (%)		5.1	56.2%	43.8%	1.7
Skewness		0.1	0.2		−2.2
Kurtosis		−0.4	−1.9		8.4
Minimum		21.0	1.0		28.0
Maximum		46.0	2.0		41.5

N = number of patients, SD = standard deviation.

**Table 2:** Comparison of obstetric and neonatal parameters in patients with and without thrombophilia.

Parameters	Thrombophilia				Chi-Square	*p*
	No		Yes			
	Mean	SD	Mean	SD		
Hospitalization Duration	6.2	3.1	6.1	3.4	1.09	0.296
Gestational Number	2.2	1.2	2.8	2.0	1.01	0.314
Parity	1.3	0.6	2.3	1.5	15.69	0.000
Neonatal Birth Weight	3116.7	584.8	2760.0	611.5	7.91	0.005

SD = standard deviation, *p* = statistical significance.

**Table 3:** Association between thrombophilia and maternal–perinatal outcomes.

Parameters		Thrombophilia				Chi-Square	*p*
		No		Yes			
		N	%	N	%		
Gestational Diabetes	No	242	87.7	25	9.1	176.3	<0.001
	Yes	9	3.3	0	0.0	-	-
Gestational Hypertension	No	226	81.9	24	8.7	163.2	<0.001
	Yes	25	9.1	1	0.4	22.15	<0.001
Mode of Delivery	Cesarean	211	76.4	17	6.2	165.1	<0.001
	Spontaneous vaginal birth	40	14.5	8	2.9	21.33	<0.001
Neonatal Sex	Male	113	40.9	12	4.3	81.60	<0.001
	Female	138	50.0	13	4.7	103.5	<0.001

N = number of patients, *p* = statistical significance.

**Table 4:** Hematological, renal, hepatic, and coagulation alterations in thrombophilic and non-thrombophilic pregnancies.

Parameters	Non-Thrombophilia		Thrombophilia		Chi-Square	*p*
	Mean	SD	Mean	SD		
WBC	11.8	2.8	11.7	3.1	0.273	0.601
HGB	14.6	29.1	11.4	1.3	8.233	0.004
HCT	35.9	3.5	34.8	3.9	1.506	0.220
PLT	233.7	62.8	253.0	89.8	1.110	0.292
Glucose	83.0	16.8	78.1	10.9	2.564	0.109
Urea	16.6	4.7	18.4	3.3	6.323	0.012
Creatinine	0.6	0.1	3.1	12.4	6.874	0.009
Uric acid	4.4	1.1	4.0	0.7	2.452	0.117
AST	22.6	36.6	24.2	28.2	1.312	0.252
ALT	21.8	45.2	19.1	18.0	0.031	0.861
Fibrinogen	528.5	118.7	479.9	87.3	4.413	0.036
INR	0.9	0.1	0.9	0.1	7.604	0.006
QUICK	10.7	1.4	10.6	0.6	0.031	0.860
APTT	26.7	2.7	25.6	1.5	5.663	0.017
N	25		226		12.68	0.001

WBC = White Blood Cell count (×10^9^/L); HGB = Hemoglobin (g/dL); HCT = Hematocrit (%); PLT = Platelet count (×10^9^/L); Glucose = Blood glucose level (mg/dL); Urea = Blood urea nitrogen (mg/dL); Creatinine = Serum creatinine (mg/dL); Uric acid = Serum uric acid (mg/dL); AST = Aspartate aminotransferase (U/L); ALT = Alanine aminotransferase (U/L); Fibrinogen = Plasma fibrinogen level (mg/dL); INR = International Normalized Ratio; QUICK = Prothrombin time percentage; APTT = Activated partial thromboplastin time (seconds), *p* = Statistical significance level, with *p* < 0.05 considered significant, N = number of patients.

**Table 5:** Associations between thrombophilia, renal dysfunction, infections, comorbidities, and treatment strategies.

Parameters		Non-Thrombophilia		Thrombophilia		Chi-Square	*p*
		N	%	N	%		
Proteinuria (mean ± SD)		18.4 ± 37.8		9.1 ± 16.0		12.57	0.000
UTIs	No	246	89.1	25	9.1	180.1	0.001
	Yes	5	1.8	0	0.0	-	-
Blood Transfusion	No	247	89.5	25	9.1	181.1	0.001
	Yes	4	1.4	0	0.0	-	-
Complication	No	239	86.6	24	8.7	175.7	0.000
	Yes	12	4.3	1	0.4	9.308	0.002
Vaginal Infections	No	126	45.7	22	8.0	73.08	0.001
	*Candida* sp.	48	17.4	1	0.4	45.08	0.001
	*E. coli*	21	7.6	2	0.7	15.69	0.001
	*Streptococcus* sp.	5	1.8	0	0.0	-	-
	*Klebsiella* sp.	6	2.2	0	0.0	-	-
	Other	19	6.9	0	0.0	-	-
	Multiple infections	26	9.4	0	0.0	-	-
Associated Pathologies (mean ± SD)		0.3 ± 0.6		0.1 ± 0.3		4.896	0.027
Treatment	No	0	0.0	25	9.1	-	-
	Enoxaparin	192	69.6	0	0.0	-	-
	Aspirin	27	9.8	0	0.0	-	-
	Enoxaparin +aspirin	32	11.6	0	0.0	-	-
N		25		251			

UTIs = urinary tract infections, N = number of patients, *p* = Statistical significance level, with *p* < 0.05 considered significant.

**Table 6:** Neonatal outcomes in thrombophilia and non-thrombophilia pregnancies: Apgar scores and NICU admissions.

Parameters		Thrombophilia				Chi-Square	*p*
		No		Yes			
		N	%	N	%		
Apgar 1 min (mean ± SD)		8.6 ± 1.4		8.1 ± 2.2		2.686	0.101
Apgar 5 min (mean ± SD)		9.6 ± 1.5		9.1 ± 2.2		2.280	0.131
NICU	No	247	89.5	21	7.6	190.5	0.000
	Yes	4	1.4	4	1.4	1.000	0.317

N = number of patients, NICU = Neonatal Intensive Care Unit, *p* = Statistical significance level, with *p* < 0.05 considered significant.

**Table 7:** Correlation between maternal clinical parameters and neonatal apgar scores.

Pearson Correlation		Apgar 1	Apgar 2
Gestational Age	r	0.568 **	0.561 **
	*p*	0.000	0.000
Birth Weight	r	0.500 **	0.496 **
	*p*	0.000	0.000
INR	r	−0.239 **	−0.214 **
	*p*	0.000	0.001
QUICK	r	−0.217 **	−0.195 **
	*p*	0.001	0.002
APTT	r	−0.283 **	−0.262 **
	*p*	0.000	0.000
Glucose	r	−0.121	−0.125 *
	*p*	0.056	0.048
Proteinuria	r	−0.261 **	−0.245 **
	*p*	0.000	0.000
	N	251	

N = number of patients, r = Pearson coefficient, INR = International Normalized Ratio; QUICK = Prothrombin time percentage; APTT = Activated partial thromboplastin time (seconds), *p* = Statistical significance level, with *p* < 0.05 considered significant, ** = Correlation is significant at the 0.01 level (2-tailed), * = Correlation is significant at the 0.05 level (2-tailed).

## Data Availability

All the data processed in this article are part of the research for a doctoral thesis, being archived in the aesthetic medical office, where the interventions were performed.
